# The Ugly Word

**Published:** 1920-07-10

**Authors:** 


### The Ugly Word.

### Mr. Galsworthy and Innocent Victims.

THE controversy over the "Bastardy Bill" now before Parliament continues, and the latest to enter the list of disputants is Mr. John Galsworthy, who has written a letter to the Press arguing from the fundamental basis of the position of the child. In this respect he takes the view which has been stated in these columns, and he approaches the subject with that large-hearted sympathy and clear thinking which characterise all his writings. The Bill has been attacked for its interference with the unmarried mother, and it has been attacked because of its menace to the putative father. It should be considered first and foremost in relation to its intention to remove injustice from the child, and Mr. Galsworthy starts with a strong attack on the very name "bastard," which is itself an insult. It is a disagreeable and ugly word, but we hardly think the suggested substitution of "nature child" is any better. It sounds pleasanter, but it implies the same thing, and that is what matters. It is not the name that counts, but the fatuous smugness and cant which allow the fact of bastard birth to be any sort of stigma on the unfortunate child. It is shamefully unjust, and Mr. Galsworthy with scornful irony proposes the introduction into the Bill of the following clause:—

"It shall be an offence punishable by fine or imprisonment for any person to call any natural child a 'bastard' or otherwise inflict indignity upon such child because of the circumstances of its birth, which, notwithstanding any Act or proviso to the contrary, are hereby declared to be beyond its control."

That is a point that must be rubbed in vigorously for the benefit of those contemptible people who make nature children the object of their narrow-minded contempt.

Some of the illogical sections of the Bill are examined by Mr. Galsworthy with exemplary logic, and especially the clause which makes a child legitimate if the parents subsequently remarry, "provided that at the time of the birth of the child, the parents might have lawfully intermarried." Any child, that is, born of parents, one of whom was married to some third party at the time of the child's birth, is still to be debarred from legitimacy though its parents afterwards become married to each other. Children born in these circumstances are (Mr. Galsworthy points out) just as innocent and just as helpless as children born of parents who were marriageable at the time of their birth, and to debar them from subsequent legitimacy is to debar at least half the illegitimate children in this country, "among them the thousands born of the irregular unions which most naturally follow that demonic invention—the judicial separation. If there ever comes in this quaint country an adequate reform of our preposterous divorce laws, a reform which enables, for instance, the judicially separated to free themselves and marry their fresh partners, their children already born will remain 'bastards' under this Bill." It has obviously been included in deterrence of illegal unions. But Mr. Galsworthy asserts that it will not deter illegal unions.

The plain truth is that in divorce reform, and measures like this Bill, the facts must be squarely faced. It is no use keeping one's head in the clouds and soaring in moral mists which have no relation to what is actually the state of things on the firm earth. It is possible to stress "moral argument" to the point of immorality, and in any case the potter has to mould with clay. If he cannot produce a thing of beauty let him at least do the best he can with the material at his disposal and not cry for the moon to be mixed up with the ingredients. It is a queer morality which makes thousands miserable, and inflicts widespread injustice with an impartial hand. What is more to the point is that the people of this modern world will not stand it.

